# Diaqua­bis­(4-hy­droxy-5-nitro­pyridine-2-carboxyl­ato-κ^2^ *N* ^1^,*O* ^2^)copper(II)

**DOI:** 10.1107/S1600536811052949

**Published:** 2011-12-14

**Authors:** Fengjuan Shi, Jiguang Deng, Hongxing Dai

**Affiliations:** a Laboratory of Catalysis Chemistry and Nanoscience, Department of Chemistry and Chemical Engineering, College of Environmental and Energy Engineering, Beijing University of Technology, Beijing 100124, People’s Republic of China

## Abstract

In the title compound, [Cu(C_6_H_3_N_2_O_5_)_2_(H_2_O)_2_], the Cu^II^ ion, lying on an inversion center, is coordinated by two pyridine N atoms and two carboxyl­ate O atoms from symmetry-related two 4-hy­droxy-5-nitro­pyridine-2-carboxyl­ate ligands, and two water mol­ecules, forming a distorted octa­hedral geometry. In the crystal, O—H⋯O hydrogen bonds link the complex mol­ecules. One of the H atoms of the water mol­ecule is disordered over two sites of equal occupancy.

## Related literature

For complexes based on the 4-hy­droxy­lpyridine-2,6-dicarb­oxy­lic acid ligand, see: Zhao *et al.* (2006 ▶, 2009 ▶, 2011 ▶). For a similar reaction to the formation of the ligand, see: Xu *et al.* (2011 ▶).
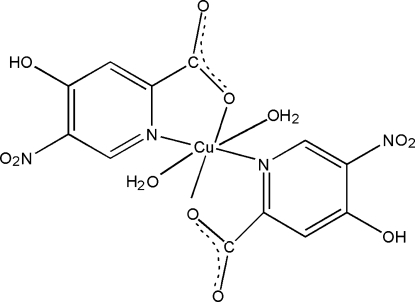


## Experimental

### 

#### Crystal data

[Cu(C_6_H_3_N_2_O_5_)_2_(H_2_O)_2_]
*M* *_r_* = 465.79
Monoclinic, 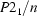

*a* = 6.5327 (7) Å
*b* = 9.7963 (10) Å
*c* = 12.2562 (12) Å
β = 102.86 (2)°
*V* = 764.68 (15) Å^3^
*Z* = 2
Mo *K*α radiation
μ = 1.52 mm^−1^
*T* = 113 K
0.20 × 0.18 × 0.10 mm

#### Data collection

Rigaku Saturn 724 CCD diffractometer
Absorption correction: multi-scan (*CrystalClear*; Rigaku, 2005 ▶) *T*_min_ = 0.752, *T*_max_ = 0.863
9563 measured reflections
1829 independent reflections
1466 reflections with *I* > 2σ(*I*)
*R*_int_ = 0.053

#### Refinement

*R*[*F* ^2^ > 2σ(*F* ^2^)] = 0.029
*wR*(*F* ^2^) = 0.077
*S* = 1.04
1829 reflections
149 parameters
6 restraints
H atoms treated by a mixture of independent and constrained refinement
Δρ_max_ = 0.39 e Å^−3^
Δρ_min_ = −0.49 e Å^−3^

### 

Data collection: *CrystalClear* (Rigaku, 2005 ▶); cell refinement: *CrystalClear*; data reduction: *CrystalClear*; program(s) used to solve structure: *SHELXS97* (Sheldrick, 2008 ▶); program(s) used to refine structure: *SHELXL97* (Sheldrick, 2008 ▶); molecular graphics: *SHELXTL* (Sheldrick, 2008 ▶); software used to prepare material for publication: *SHELXTL*.

## Supplementary Material

Crystal structure: contains datablock(s) global. DOI: 10.1107/S1600536811052949/hy2493sup1.cif

Additional supplementary materials:  crystallographic information; 3D view; checkCIF report

## Figures and Tables

**Table 1:** Hydrogen-bond geometry (Å, °)

*D*—H⋯*A*	*D*—H	H⋯*A*	*D*⋯*A*	*D*—H⋯*A*
O3—H3⋯O2^i^	0.79 (3)	1.79 (3)	2.544 (2)	160 (3)
O6—H6*A*⋯O2^ii^	0.84 (1)	2.28 (2)	3.014 (2)	146 (3)
O6—H6*B*⋯O6^iii^	0.85 (1)	2.00 (1)	2.836 (3)	170 (5)
O6—H6*C*⋯O3^iv^	0.85 (1)	2.49 (3)	3.109 (2)	130 (3)

Symmetry codes: (i) 
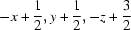
; (ii) 
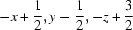
; (iii) 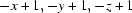; (iv) 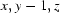.

**Fig. 1.**
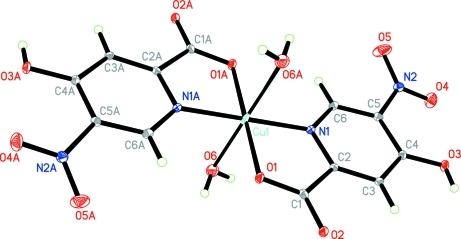
Molecular structure of the title compound. Displacement ellipsoids are drawn at the 30% probability level. One of the disordered H atom sites on water molecule is not shown. [Symmetry code: (A) -x, 1-y, 1-z.]

## supplementary crystallographic information

### Comment

Carboxylate ligands play an important role in constructing novel metal-organic frameworks (MOFs) in coordination chemistry. Especially, a large number of MOFs based on pyridyl dicarboxylic acid ligands containing N- and O-donors with multi-connecting ability have been constructed. 4-Hydroxyl-pyridine-2,6-dicarboxylic acid has been widely used in the construction of high-dimensional structures with large pores. It usually adopts diverse coordination binding modes such as chelating to one metal center, bridging bidentate in *syn*–*syn* or *syn*–*anti* configuration to two or three metal centers. A systematic study of 3d–4f, 4d–4f and 3d–4d–4f complexes based on 4-hydroxyl-pyridine-2,6-dicarboxylic acid ligand has been undertaken (Zhao *et al.*, 2006, 2009, 2011). However, to the best of our knowledge, no reports on the synthesis of the title compound have been seen in literature. In this paper, we report the synthesis and crystal structure of the title compound using a hydrothermal method with 4-hydroxyl-pyridine-2,6-dicarboxylic acid as ligand.

The title compound features a 3-nitro-4-hydroxyl-pyridine-6-carboxylate ligand, which was in situ generated by decarboxylation and nitration of 4-hydroxyl-pyridine-2,6-dicarboxylic acid under the hydrothermal conditions. A similar reaction has been reported (Xu *et al.*, 2011). Structure analysis shows that the Cu^II^ ion is centrosymmetrically coordinated by two N atoms [Cu—N = 1.9685 (16) Å] and two carboxylate O atoms [Cu—O = 1.9667 (15) Å] from two 3-nitro-4-hydroxyl-pyridine-6-carboxylate ligands and two water molecules [Cu—O = 2.479 (2) Å], forming a distorted octahedral geometry, as shown in Fig. 1. O—H···O hydrogen bonds link the complex molecules (Table 1).

### Experimental

A mixture of 4-hydroxyl-pyridine-2,6-dicarboxylic acid (366 mg, 2.0 mmol), copper nitrate trihydrate (242 mg, 1.0 mmol), lanthanide nitrate hexahydrate (Ln = Eu, Sm, Pr, Tb) (1.0 mmol) and deionized water (10 ml) was placed in a 25 ml Teflon-lined steel autoclave, which was kept at 433 K for 3 days. The resuling blue prismatic crystals suitable for X-ray diffraction experiment were collected after washing with deionized water and diethyl ether (yield: 43% based on the mass of copper nitrate trihydrate).

### Refinement

H atoms bound to C atoms were positioned geometrically and refined as riding atoms, with C—H = 0.95 Å and with *U*_iso_(H) = 1.2*U*_eq_(C). H atoms bound to O atoms were located from a difference Fourier map and refined isotropically. One of H atoms of the water molecule is disordered over two sites with equal occupancy factors.

### Crystal data

**Table:**

[Cu(C_6_H_3_N_2_O_5_)_2_(H_2_O)_2_]	*F*(000) = 470
*M**_r_* = 465.79	*D*_x_ = 2.023 Mg m^−^^3^
Monoclinic, *P*2_1_/*n*	Mo *K*α radiation, λ = 0.71073 Å
Hall symbol: -P 2yn	Cell parameters from 3085 reflections
*a* = 6.5327 (7) Å	θ = 2.1–27.9°
*b* = 9.7963 (10) Å	µ = 1.52 mm^−^^1^
*c* = 12.2562 (12) Å	*T* = 113 K
β = 102.86 (2)°	Prism, colorless
*V* = 764.68 (15) Å^3^	0.20 × 0.18 × 0.10 mm
*Z* = 2	

### Data collection

**Table:**

Rigaku Saturn 724 CCD diffractometer	1829 independent reflections
Radiation source: rotating anode	1466 reflections with *I* > 2σ(*I*)
multilayer	*R*_int_ = 0.053
Detector resolution: 14.22 pixels mm^-1^	θ_max_ = 27.9°, θ_min_ = 2.7°
ω scans	*h* = −8→7
Absorption correction: multi-scan (*CrystalClear*; Rigaku, 2005)	*k* = −12→12
*T*_min_ = 0.752, *T*_max_ = 0.863	*l* = −15→16
9563 measured reflections	

### Refinement

**Table:**

Refinement on *F*^2^	Primary atom site location: structure-invariant direct methods
Least-squares matrix: full	Secondary atom site location: difference Fourier map
*R*[*F*^2^ > 2σ(*F*^2^)] = 0.029	Hydrogen site location: inferred from neighbouring sites
*wR*(*F*^2^) = 0.077	H atoms treated by a mixture of independent and constrained refinement
*S* = 1.04	*w* = 1/[σ^2^(*F*_o_^2^) + (0.036*P*)^2^] where *P* = (*F*_o_^2^ + 2*F*_c_^2^)/3
1829 reflections	(Δ/σ)_max_ < 0.001
149 parameters	Δρ_max_ = 0.39 e Å^−^^3^
6 restraints	Δρ_min_ = −0.49 e Å^−^^3^

### Special details

**Table:**

Experimental. Rigaku *CrystalClear*-SM Expert 2.0 r2
Geometry. All e.s.d.'s (except the e.s.d. in the dihedral angle between two l.s. planes) are estimated using the full covariance matrix. The cell e.s.d.'s are taken into account individually in the estimation of e.s.d.'s in distances, angles and torsion angles; correlations between e.s.d.'s in cell parameters are only used when they are defined by crystal symmetry. An approximate (isotropic) treatment of cell e.s.d.'s is used for estimating e.s.d.'s involving l.s. planes.
Refinement. Refinement of *F*^2^ against ALL reflections. The weighted *R*-factor *wR* and goodness of fit *S* are based on *F*^2^, conventional *R*-factors *R* are based on *F*, with *F* set to zero for negative *F*^2^. The threshold expression of *F*^2^ > σ(*F*^2^) is used only for calculating *R*-factors(gt) *etc*. and is not relevant to the choice of reflections for refinement. *R*-factors based on *F*^2^ are statistically about twice as large as those based on *F*, and *R*- factors based on ALL data will be even larger.

### Fractional atomic coordinates and isotropic or equivalent isotropic displacement parameters (Å^2^)

**Table:**

	*x*	*y*	*z*	*U*_iso_*/*U*_eq_	Occ. (<1)
Cu1	0.0000	0.5000	0.5000	0.01573 (13)	
O1	0.0540 (2)	0.53885 (14)	0.66132 (12)	0.0145 (3)	
O2	0.1475 (2)	0.70789 (14)	0.78407 (11)	0.0150 (3)	
O3	0.3093 (2)	1.08681 (14)	0.52856 (13)	0.0144 (3)	
H3	0.331 (5)	1.107 (3)	0.592 (2)	0.058 (11)*	
O4	0.2830 (3)	1.07679 (17)	0.31323 (14)	0.0291 (4)	
O5	0.0819 (3)	0.9216 (2)	0.22215 (13)	0.0408 (5)	
O6	0.3578 (3)	0.40247 (18)	0.52615 (15)	0.0253 (4)	
H6A	0.371 (5)	0.381 (3)	0.5936 (10)	0.060 (11)*	
H6B	0.454 (7)	0.455 (5)	0.516 (3)	0.06 (2)*	0.50
H6C	0.348 (10)	0.332 (3)	0.484 (3)	0.09 (3)*	0.50
N1	0.1030 (3)	0.68775 (16)	0.49202 (13)	0.0113 (4)	
C1	0.1142 (3)	0.6591 (2)	0.68823 (16)	0.0121 (4)	
C2	0.1493 (3)	0.7498 (2)	0.59364 (16)	0.0108 (4)	
C3	0.2222 (3)	0.8799 (2)	0.61022 (16)	0.0109 (4)	
H3A	0.2576	0.9169	0.6837	0.013*	
C4	0.2450 (3)	0.9596 (2)	0.51800 (17)	0.0106 (4)	
C5	0.1881 (3)	0.8957 (2)	0.41301 (16)	0.0114 (4)	
C6	0.1219 (3)	0.7610 (2)	0.40323 (16)	0.0119 (4)	
H6	0.0891	0.7197	0.3313	0.014*	
N2	0.1874 (3)	0.97025 (17)	0.30795 (15)	0.0150 (4)	

### Atomic displacement parameters (Å^2^)

**Table:**

	*U*^11^	*U*^22^	*U*^33^	*U*^12^	*U*^13^	*U*^23^
Cu1	0.0276 (2)	0.00876 (18)	0.0110 (2)	−0.00692 (15)	0.00460 (15)	−0.00212 (14)
O1	0.0212 (8)	0.0095 (7)	0.0123 (8)	−0.0040 (6)	0.0028 (6)	0.0002 (5)
O2	0.0251 (8)	0.0103 (7)	0.0088 (7)	−0.0004 (6)	0.0021 (6)	−0.0006 (6)
O3	0.0201 (8)	0.0090 (7)	0.0146 (8)	−0.0040 (6)	0.0052 (7)	−0.0001 (6)
O4	0.0442 (11)	0.0199 (9)	0.0253 (9)	−0.0091 (8)	0.0124 (8)	0.0029 (7)
O5	0.0523 (13)	0.0497 (12)	0.0151 (9)	−0.0240 (10)	−0.0036 (9)	0.0070 (8)
O6	0.0271 (10)	0.0238 (9)	0.0234 (10)	−0.0057 (8)	0.0019 (8)	0.0029 (8)
N1	0.0125 (9)	0.0104 (8)	0.0100 (8)	−0.0010 (7)	0.0004 (7)	−0.0013 (6)
C1	0.0107 (10)	0.0107 (10)	0.0149 (11)	0.0005 (8)	0.0027 (8)	0.0031 (8)
C2	0.0123 (10)	0.0102 (9)	0.0091 (10)	0.0006 (8)	0.0006 (8)	0.0012 (7)
C3	0.0109 (10)	0.0116 (10)	0.0093 (10)	0.0000 (8)	0.0007 (8)	−0.0011 (7)
C4	0.0077 (9)	0.0100 (9)	0.0145 (10)	0.0007 (7)	0.0030 (8)	−0.0003 (7)
C5	0.0104 (10)	0.0139 (10)	0.0105 (10)	−0.0004 (8)	0.0034 (8)	0.0030 (8)
C6	0.0117 (11)	0.0149 (10)	0.0099 (10)	−0.0002 (8)	0.0036 (8)	−0.0028 (8)
N2	0.0150 (9)	0.0165 (9)	0.0136 (9)	−0.0015 (7)	0.0033 (8)	0.0011 (7)

### Geometric parameters (Å, °)

**Table:**

Cu1—N1	1.9685 (16)	O6—H6C	0.85 (1)
Cu1—O1	1.9667 (15)	N1—C6	1.332 (2)
Cu1—O6	2.479 (2)	N1—C2	1.358 (2)
O1—C1	1.263 (2)	C1—C2	1.518 (3)
O2—C1	1.242 (2)	C2—C3	1.360 (3)
O3—C4	1.312 (2)	C3—C4	1.409 (3)
O3—H3	0.79 (3)	C3—H3A	0.9500
O4—N2	1.211 (2)	C4—C5	1.405 (3)
O5—N2	1.219 (2)	C5—C6	1.385 (3)
O6—H6A	0.84 (1)	C5—N2	1.480 (2)
O6—H6B	0.85 (1)	C6—H6	0.9500
			
O1—Cu1—O1^i^	180.0	O2—C1—C2	118.24 (17)
O1—Cu1—N1	83.24 (6)	O1—C1—C2	115.97 (17)
O1^i^—Cu1—N1	96.76 (6)	N1—C2—C3	123.75 (18)
O1—Cu1—N1^i^	96.76 (6)	N1—C2—C1	113.45 (17)
O1^i^—Cu1—N1^i^	83.24 (6)	C3—C2—C1	122.80 (18)
N1—Cu1—N1^i^	180.0	C2—C3—C4	119.73 (18)
O1—Cu1—O6	89.52 (6)	C2—C3—H3A	120.1
O1—Cu1—O6^i^	90.48 (6)	C4—C3—H3A	120.1
N1—Cu1—O6^i^	87.50 (7)	O3—C4—C5	121.85 (18)
O6—Cu1—N1	92.50 (7)	O3—C4—C3	122.42 (18)
O6—Cu1—O6^i^	180.00	C5—C4—C3	115.70 (18)
C1—O1—Cu1	114.76 (13)	C6—C5—C4	121.20 (18)
C4—O3—H3	109 (2)	C6—C5—N2	117.04 (17)
H6A—O6—H6B	112.7 (17)	C4—C5—N2	121.71 (17)
H6A—O6—H6C	111.5 (17)	N1—C6—C5	121.74 (18)
H6B—O6—H6C	111.2 (17)	N1—C6—H6	119.1
C6—N1—C2	117.79 (17)	C5—C6—H6	119.1
C6—N1—Cu1	129.69 (14)	O4—N2—O5	124.71 (18)
C2—N1—Cu1	112.42 (13)	O4—N2—C5	118.54 (17)
O2—C1—O1	125.79 (19)	O5—N2—C5	116.68 (17)
			
N1—Cu1—O1—C1	3.72 (14)	N1—C2—C3—C4	2.6 (3)
N1^i^—Cu1—O1—C1	−176.28 (14)	C1—C2—C3—C4	−178.09 (18)
O1—Cu1—N1—C6	−178.60 (18)	C2—C3—C4—O3	178.19 (18)
O1^i^—Cu1—N1—C6	1.40 (18)	C2—C3—C4—C5	0.1 (3)
O1—Cu1—N1—C2	−2.41 (13)	O3—C4—C5—C6	179.52 (18)
O1^i^—Cu1—N1—C2	177.59 (13)	C3—C4—C5—C6	−2.4 (3)
Cu1—O1—C1—O2	176.32 (16)	O3—C4—C5—N2	−3.1 (3)
Cu1—O1—C1—C2	−4.1 (2)	C3—C4—C5—N2	175.00 (17)
C6—N1—C2—C3	−3.0 (3)	C2—N1—C6—C5	0.5 (3)
Cu1—N1—C2—C3	−179.66 (16)	Cu1—N1—C6—C5	176.56 (14)
C6—N1—C2—C1	177.69 (16)	C4—C5—C6—N1	2.1 (3)
Cu1—N1—C2—C1	1.0 (2)	N2—C5—C6—N1	−175.38 (17)
O2—C1—C2—N1	−178.33 (17)	C6—C5—N2—O4	−165.03 (19)
O1—C1—C2—N1	2.1 (3)	C4—C5—N2—O4	17.5 (3)
O2—C1—C2—C3	2.3 (3)	C6—C5—N2—O5	18.0 (3)
O1—C1—C2—C3	−177.27 (18)	C4—C5—N2—O5	−159.5 (2)

Symmetry codes: (i) −*x*, −*y*+1, −*z*+1.

### Hydrogen-bond geometry (Å, °)

**Table:**

*D*—H···*A*	*D*—H	H···*A*	*D*···*A*	*D*—H···*A*
O3—H3···O2^ii^	0.79 (3)	1.79 (3)	2.544 (2)	160 (3)
O6—H6A···O2^iii^	0.84 (1)	2.28 (2)	3.014 (2)	146 (3)
O6—H6B···O6^iv^	0.85 (1)	2.00 (1)	2.836 (3)	170 (5)
O6—H6C···O3^v^	0.85 (1)	2.49 (3)	3.109 (2)	130 (3)

Symmetry codes: (ii) −*x*+1/2, *y*+1/2, −*z*+3/2; (iii) −*x*+1/2, *y*−1/2, −*z*+3/2; (iv) −*x*+1, −*y*+1, −*z*+1; (v) *x*, *y*−1, *z*.
